# Minimally Invasive Decompression and Physiotherapy for Lumbar Spinal Stenosis in Geriatric Patients

**DOI:** 10.7759/cureus.2785

**Published:** 2018-06-11

**Authors:** Haydn Hoffman, Shelley S Bennett, Charles H Li, Piia Haakana, Daniel C Lu

**Affiliations:** 1 Department of Neurosurgery, University of California Los Angeles; 2 Department of Physical Therapy, University of California, Los Angeles; 3 Department of Neurosurgery, University of California, Los Angeles

**Keywords:** spinal stenosis, lumbar decompression, postoperative rehabilitation

## Abstract

Background

Lumbar spinal stenosis (LSS) is the most common indication for spine surgery among the geriatric population. Although decompressive surgery is effective, older patients do not benefit as much as younger patients, and they are frequently excluded from studies assessing postoperative physiotherapy. We sought to evaluate the long-term outcomes after surgery when a novel postoperative physiotherapy regimen was included.

Methods

We performed a retrospective review of patients with LSS greater than 70 years old who underwent lumbar decompressive surgery by the senior author over the past five years. We evaluated patients who participated in a novel postoperative physiotherapy regimen involving four phases of rehabilitation aimed at progressively independent ambulation. The visual analog scale (VAS), lower extremity motor strength, and functional independence measure (FIM) were collected preoperatively and after physiotherapy to measure outcomes.

Results

Ten consecutive patients with an average age of 83 years (range: 71 – 96) met the inclusion criteria. Nine patients underwent minimally invasive laminotomies at L4-L5 and one underwent a laminotomy at L3-L4. The average follow-up time was 41.9 months. The preoperative mean VAS was 7.35, and at the end of the study, it was 1.7 (p = 0.005). Three of the four patients with preoperative motor deficits improved. The median transfer and locomotion subscores of the FIM were six preoperatively and increased to seven postoperatively. Neither of these improvements was significant.

Conclusions

Patients older than 70 years undergoing decompressive surgery and a novel postoperative physiotherapy regimen experienced significant reductions in pain. Independence also increased; however, this did not reach statistical significance.

## Introduction

Degenerative lumbar spinal stenosis (LSS) is one of the most common etiologies of back pain and radiculopathy affecting the geriatric population. Given the aging population of the United States, there has been a 23% increase in surgical operations performed for LSS [1]. In recent years, there has been an increased focus on surgical interventions for LSS in older patients spurred by advances in minimally invasive techniques that minimize the morbidity of back surgery [2-4]. There is limited outcome data on postoperative physiotherapy for LSS in the geriatric population, however [5]. Recent data suggest that postoperative rehabilitation following decompressive surgery is effective at reducing pain and improving quality of life and functional status, but geriatric patients have been largely excluded from these studies [6-7]. Furthermore, specific rehabilitation regimens for the geriatric population following surgery for LSS have not been described, and the nature of those for the general population is heterogeneous [8].

Given the lack of data regarding postoperative rehabilitation after lumbar decompressive surgery in geriatric patients, we sought to evaluate the efficacy and feasibility of a novel physiotherapy regimen for this population. The proposed regimen included four phases aimed at restoring ambulation: (1) extremity and core muscle conditioning, (2) balance and stance training, (3) cardiovascular conditioning, and (4) sit-to-stand training. We were interested in the pain and functional status of these patients following completion of rehabilitation. This data will guide surgeons and physical therapists in the postoperative management of older patients with LSS.

## Materials and methods

Patient recruitment

After obtaining institutional review board approval, patients with LSS presenting to the clinic of the senior author were considered for inclusion in the study. Diagnosis of LSS was confirmed by findings on magnetic resonance imaging and clinical correlation of the patient’s symptoms. Inclusion criteria included age > 70 years, symptoms of radiculopathy and/or neurogenic claudication, radiographic evidence of spinal stenosis, and medical clearance for surgery. Exclusion criteria included a history of prior lumbar spine surgery, spondylolisthesis, scoliosis, and orthopedic or peripheral neurologic comorbidities that could have confounded ambulation status. All patients underwent minimally invasive laminotomy by the senior author according to a technique previously described (Video 1) [9]. This surgical approach was performed through a small 1.5 cm incision. In most cases, patients were able to go home on the day of their procedure.

**Video 1:** Minimally invasive laminotomy [9]

Physiotherapy regimen

The physiotherapy was designed and performed individually and face-to-face by a single physical therapist in order to eliminate potential inter-rater disagreement. The physiotherapy consisted of a weekly 90 minute adaptive, graded regimen that was divided into four phases to address specific deficits with the final goal of restoring independent standing and ambulatory abilities (Table 1). Adherence to therapy sessions was assessed by the physical therapist and all participants adhered to this schedule.

**Table 1:** Phases of Rehabilitation

Phase	Stage	Recommended postoperative start date
1	Extremity and core muscle conditioning	1 week
2	Balance and stance training	1 month
3	Cardiovascular conditioning and gait training	2 months
4	Sit-to-stand training	3 months

The timing of each phase was dependent on accomplishing the tasks in the preceding phase. Phase 1 consisted of extremity and core muscle strengthening to build the necessary substrates for motor function. This began one week postoperatively and was continued for the duration of the regimen. Initially, exercises were performed in the supine position and then were advanced to the standing stationary position. Adjunctive functional electrical stimulation (FES) was carried out as described in Table 2.

**Table 2:** Phase 1 Exercises and Functional Electric Stimulation Protocol

Exercise	Performance	Goal
Supine exercises		# of repetitions
Bilateral ankle pumps	With legs elevated in bed, gently flex and extend ankles	10
Hip abduction/adduction	With patient lying on side, asked to abduct and adduct hips. Switch sides.	10
Bilateral heel slides	With legs fully extended, heels are moved up towards the buttocks as far as comfortable	10
Sitting or standing exercises		No. repetitions
Sit to stand at walker	With maximum assistance, the patient is asked to stand with support of walker	As many as possible; track repetitions over time
Bilateral heel raises	With legs flexed, the patient is asked to raise heels while seated in walker	10
Bilateral knee extension/flexion	Patient is asked to flex and extend knees while seated in walker	10
Restorator	Upper extremity strengthening machine, the patient is asked to rotate device with arms	As many as possible in 3 minutes at a speed of 4.0 with medium resistance
Ball squeezes	Rubber ball placed between patient’s legs; patient asked to flex and relax against ball resistance	10 for 3 sets
Isometric quadriceps squeezes	Patient started without resistance and eventually progressed to 2.5 lbs ankle weights for isometric quad squeezes	5 for 10 seconds each contraction
Scapular contractions	Isometric contraction and relaxation of scapular muscles	10
Bilateral marching	With assistance, the patient is asked to march in place to work on hip flexion; patient is progressed to marching with ankle weights	10
Functional electrical stimulation		Time (minutes)
Gastrocnemius	Stimulation for 40 minutes at 20-30 Hz intensity; vitals monitored	40
Gluteus maximus	Stimulation for 40 minutes at 20-30 Hz intensity; vitals monitored	40
Tibialis anterior	Stimulation for 40 minutes at 20-30 Hz intensity; vitals monitored	40
Hamstrings	Stimulation for 40 minutes at 20-30 Hz intensity; vitals monitored	40

Phase 2 was comprised of aquatic balance and stance training to accommodate the upright position. Since phase 2 began approximately one month postoperatively, surgical incisions had healed sufficiently to allow for safe exposure to water. Aquatic therapy was favored over ground exercises because it reduced axial load on the spine and had been suggested to be better tolerated in the geriatric population [10]. Phase 2 therapy sessions lasted approximately 40 minutes a day and were focused on standing and locomotion with the goal of increasing the duration of standing with minimal assistance.

Phase 3 involved cardiovascular conditioning and gait training to accommodate the cardiovascular demand of ambulation. This began with the prerequisite that the patient could stand with or without assistance for at least 10 seconds. Patients were instructed to walk in a straight line (with the assistance of a front-wheel walker as needed) with the goal of increasing the maximum distance tolerated before needing to rest.

Phase 4 involved sit-to-stance training.

Outcome measurement and data analysis

Outcome data were obtained by the physical therapist. The visual analog scale (VAS) was used to assess the severity of each patient’s pain before surgery and at the end of phase 4. When a patient had both back and leg pain, the source of pain most concerning to the patient was considered. In addition, each patient’s strength was measured before surgery and at the end of phase 4 by testing the muscles associated with each lumbar and sacral nerve root. Strength was measured on a scale from 0 to 5 as defined by the Medical Research Council Scale for Muscle Strength. The functional independence measure (FIM) is a reliable tool for evaluating a subject’s disability in the domains of self-care, sphincter management, mobility, and locomotion. It was used to evaluate each patient before surgery and at the end of phase 4. Wilcoxon signed-rank test was used to compare scores before and after surgery.

Electromyography (EMG) was performed on patient #10 at 10 and 16-month postoperative time intervals to measure his improvement over the course of sit-to-stand training. Data was collected at 2,000 Hz and subsequently processed in Matlab (Mathworks, Natick, MA). The following were sequentially applied to the data: direct current (DC) offset removal, 5th-order Butterworth bandpass filter (10 - 500 Hz), rectification, and linear envelope. The EMG signals were analyzed to compute the amplitude, timing, and duration of individual electrical bursts. Root-mean-square (RMS) EMG activity of the rectus femoris (RF), hamstrings (HAM), tibialis anterior (TA), and medial gastrocnemius (MG) muscles were calculated. EMG amplitudes were not normalized because the same patient’s data was compared six months apart and muscles were analyzed individually.

## Results

A total of 10 patients (three females, seven males) met the inclusion criteria and were included in the study. The average age was 83 years (range: 71 - 96). Additional demographic data is shown in Table 3. Six patients underwent single-level minimally invasive laminotomies at L4-L5 and one underwent a single level laminotomy at L3-L4. Three patients underwent surgery at multiple levels, as shown in Table 3. There were no intraoperative complications and no patients required reoperation. The average duration of surgery was 62 minutes (range: 38 - 120). The average follow-up time was 41.9 months (range: 24 - 60). No adverse effects from the physiotherapy were observed in the cohort.

**Table 3:** Patient Demographics F: female; M: male; L: lumbar

Patient no.	Age	Gender	Procedure levels	Follow-up time (months)
1	71	F	L4-5	60
2	82	M	L3-4	36
3	78	M	L2-3, L4-5	38
4	77	F	L4-5	34
5	92	M	L4-5	26
6	84	F	L4-5	57
7	89	M	L4-5	54
8	85	M	L4-5	50
9	76	M	L4-5, L5-S1	40
10	96	M	L3-4, L4-5	24

**Figure 1:**
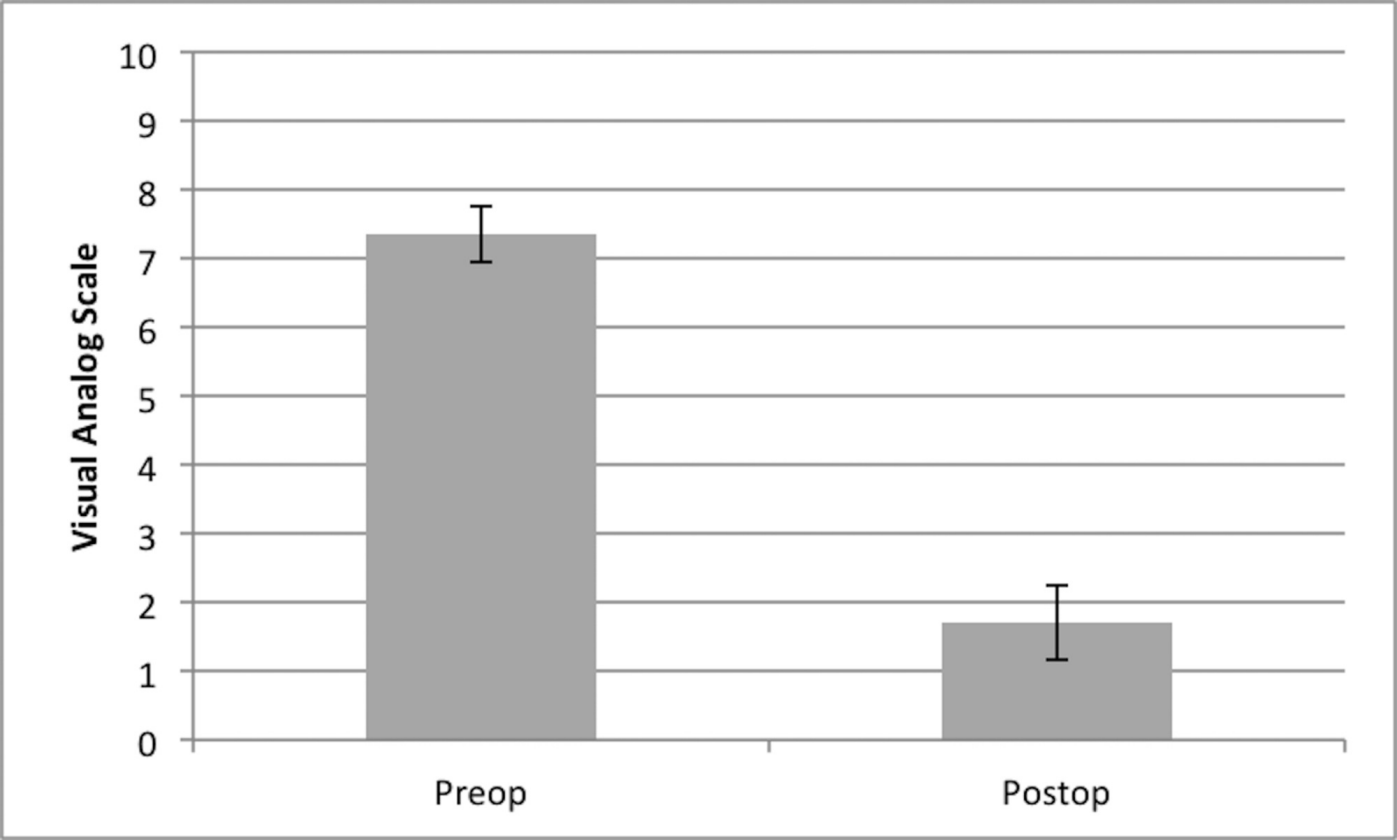
Mean visual analog scale scores decreased significantly after surgery and physiotherapy (p = 0.005) Error bars indicate standard error of the mean.

The mean VAS score for the group was 7.35 (standard deviation (SD) = 1.29) before surgery and 1.7 (SD = 1.7) at the end of the study (Figure 1). The decrease was statistically significant (p = 0.005). The mean reduction in the VAS score at the end of the study was 5.65 (SD = 1.9). Each patient experienced improvement in their VAS score after surgery.

Preoperatively, four patients had objective motor weakness in the lower extremities. Subject #4 had 4/5 strength with right-sided dorsiflexion, which returned to full strength at the end of the study. Subject #7 had 4/5 strength with right-sided knee extension, and she also returned to full strength at the end of the study. Preoperatively, Subject #9 had 3/5 strength with left-sided extensor hallucis longus, and this was unchanged at the end of the study. Finally, Subject #10 had 1/5 strength preoperatively with right-sided dorsiflexion, and this improved to 4/5 at the end of the study. His left-sided dorsiflexion was 3/5 preoperatively, and this improved to full strength. None of the patients experienced a decrease in strength after surgery.

**Figure 2:**
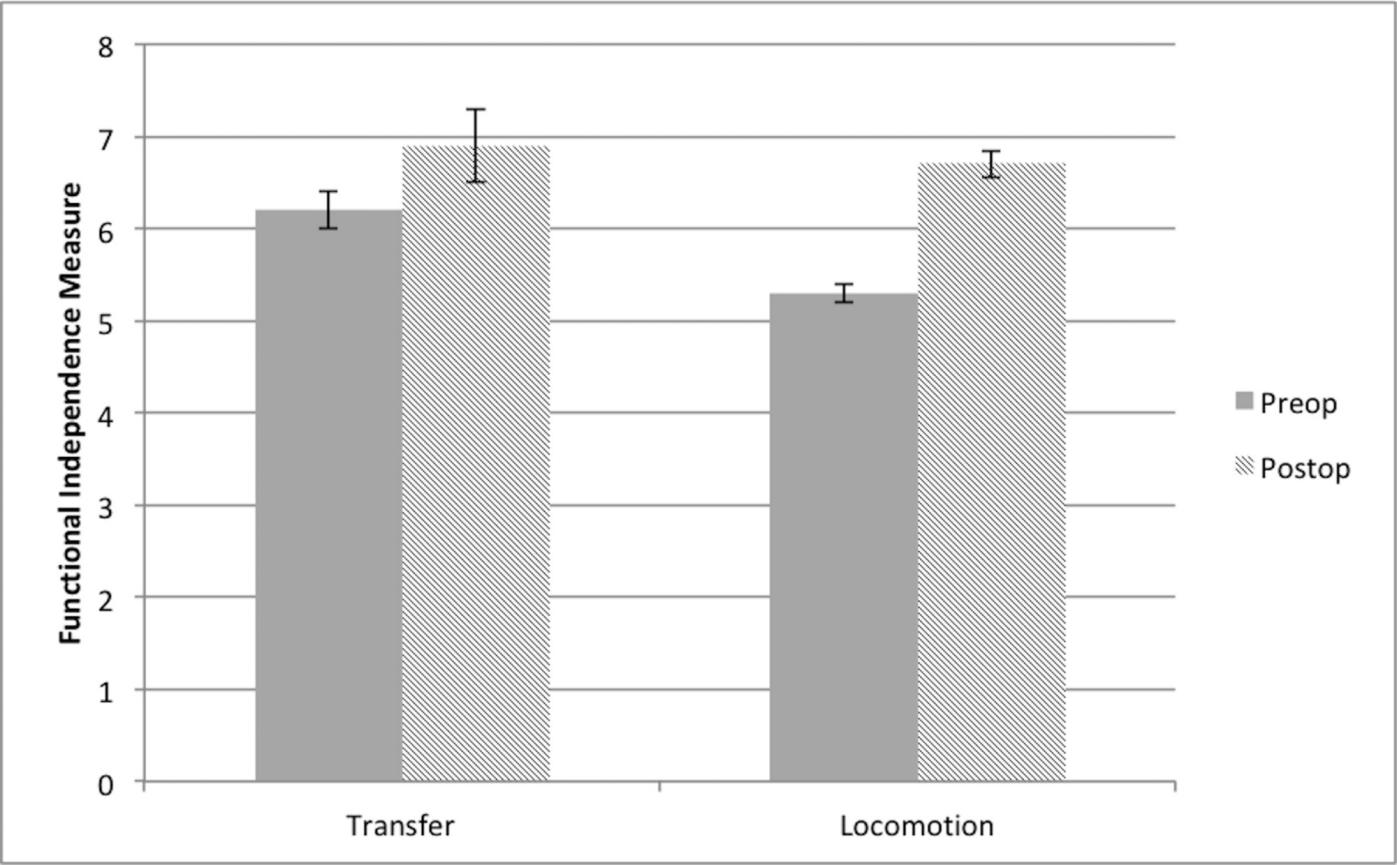
Functional independence measure scores Mean functional independence measure scores for transfer and locomotion domains increased after surgery but this was not statistically significant. Error bars indicate standard error of the mean

FIM scores were available for nine of the 10 patients. The patient with missing FIM data was excluded from this analysis. Preoperative deficits were only in the domains of transfers and locomotion and were compared to postoperative scores in Figure 2. The median preoperative transfer score was 6 (range: 5 - 7) and increased to 7 (range: 6 - 7) at the end of the study. The median preoperative locomotion score was 6 (range: 3 - 7) and increased to 7 (range: 6 - 7). Neither of these improvements was statistically significant. Both patients who required assistance ambulating preoperatively (scores of 3 and 4) no longer required assistance at the end of the study (both increased to scores of 6).

**Figure 3:**
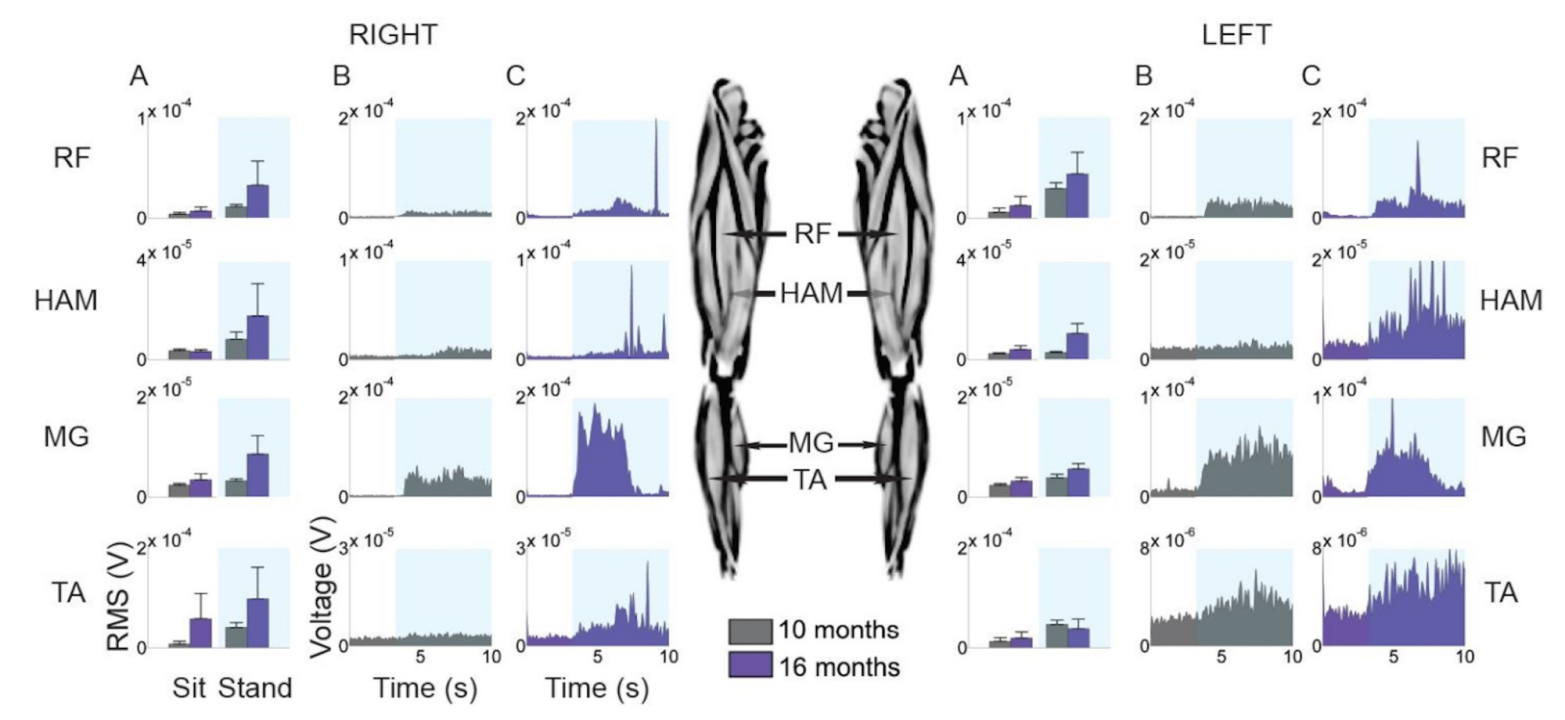
EMG recordings during sit-to-stand training for patient #10 at 10 months and 16 months postoperatively. Root-mean-square value with position (a) and absolute amplitude over the duration of the activity at 10 months (b) and 16 months (c) are provided. EMG: electromyography; RF: rectus femoris; HAM: hamstrings; TA: tibialis anterior; MG: medial gastrocnemius; RMS: root-mean-square.

EMG recordings were available for patient #10 at 10 and 16 months postoperatively. At the latter time point, there were increases in the RMS amplitudes throughout the right and left muscle groups (except the left TA) when the patient stood up (Figure 3A). The improvement was more pronounced on the patient’s right side, corresponding to his deficit prior to surgery. Additionally, the frequency of EMG activity was measured during the patient’s sit-to-stand training and a similar increase in amplitudes was observed at the 16-month time point (Figure 3B-C). This was most evident in the right MG. 

## Discussion

LSS is the most common indication for spinal surgery in patients older than 65 years [3]. Surgery should not be withheld from this population based on age alone because it has been shown to be superior to non-operative therapy for improving pain, disability, and quality of life [11]. Given that almost 25% of patients undergoing decompression for LSS do not benefit and require reoperation [12], maximizing the benefits of surgery is important. This is even more crucial in the older population, which is more vulnerable to postoperative complications, comorbidity, and increasingly advanced pathology. Rinh et al. found that octogenarians did not improve as much as younger patients with regard to pain and self-rated progress after surgery for LSS [11]. In addition, the postoperative complication rate for older patients undergoing lumbar spine surgery has been reported to be higher than those less than 65 years old [13-16]. In those older than 75 years, the wound complication rate has been reported to be as high as 28%, and the systemic complication rate as high as 18% [17]. When obesity is present, the reoperation rate is as high as 9.6% [18]. Thus, the geriatric population merits special attention to improve outcomes.

In the general population, postoperative physiotherapy for LSS has frequently been shown to be helpful in the recovery of strength and movement, as well as the resolution of pain [7, 19]. Depending on the primary outcome measure and follow-up time, however, the benefit is inconsistent. Mannion et al. performed a randomized controlled trial of 159 patients who underwent decompressive surgery for LSS with or without supervised postoperative physiotherapy [20]. The changes in pain and disability levels up to 24 months after surgery were not different between the groups. Given the discrepancy in improvement after decompression for LSS in geriatric patients and the lack of evidence regarding postoperative physiotherapy in this population, we sought to evaluate the outcomes of a physiotherapy regimen that was initiated after surgery for LSS.

Significant improvement in pain was noted in our study group. The mean VAS score decreased by a total of 5.65 points after surgery and physiotherapy. This compares favorably to large cohorts of geriatric patients receiving decompression without physiotherapy. Rosen et al. showed a 3.5 and 3.4 point decrease in back and leg pain, respectively, in 50 patients older than 75 years [21]. In a retrospective series of 125 elderly patients followed five years postoperatively, the average reduction in the VAS score was 5.1 points [22]. Without a randomized trial, it is not possible to determine whether the improvements seen in our series were augmented by physiotherapy or simply the effect of surgery. Regardless, it is encouraging that our series experienced such a high level of improvement. 

We did not observe significant differences in FIM scores after surgery and physiotherapy. This was likely due to the small sample size and the fact that preoperatively most patients only had mild reductions in their independence. Both patients who required assistance ambulating preoperatively demonstrated independence at the end of phase 4, however. All patients showed improvement in at least one domain at the end of the study. The domains of self-care and sphincter control were omitted because none of the patients endorsed limitations in performing these activities. Similarly, preoperative deficits in strength were minor and heterogeneous, precluding statistical analysis. Only one patient who had a deficit did not improve at the end of the study, likely because this was longstanding over several years.

Limited data exist to guide the development of a physiotherapy regimen for geriatric patients with LSS, and the data that does exist is largely derived from non-surgical populations. Furthermore, no standard of care exists for the delivery of physiotherapy for LSS [23], and when regimens are evaluated, the actual amount of therapy delivered is frequently not standardized or adequately described [24-25]. Likewise, the modalities applied in the community are heterogeneous [26]. The phases of rehabilitation in our regimen were designed to represent an incremental progression of ambulatory ability because ambulation is a primary concern of older patients with neurogenic claudication [23]. Strengthening and aerobic conditioning are central components of each phase, which have been shown to be effective for reducing pain and disability in patients with LSS [27]. The phases were designed such that it would have been difficult to achieve competence in a higher phase without having accomplished the preceding phases. For example, it is difficult to achieve stepping and gait (phase 3) without having muscle strength (phase 1) and balance (phase 2). Dividing ambulation into distinct goals can motivate subjects’ participation. Compliance is likely to be more readily obtained if small milestones (i.e., phases) are successfully obtained over time [28], and we observed this in our study group. This is especially pertinent to the geriatric population, in which deconditioning and comorbidity may limit participation. A randomized trial is needed to assess the efficacy of this approach compared to other modes of physiotherapy.

This study is limited by the small sample receiving our intervention, which was not sufficient to detect small improvements in functional independence. Regardless, we were able to demonstrate a statistically significant improvement in pain that compared favorably to other published data [21-22]. Additionally, we did not include a control group (i.e., surgery without postoperative rehabilitation) to elucidate what amount of improvement, if any, was attributable to the physiotherapy. The generalizability of this study is limited by the fact that interventions were carried out by a single neurosurgeon and physical therapist at a single institution. This study does, however, suggest that postoperative rehabilitation is feasible in an older population with LSS and provides a regimen that can be tolerated with good compliance. A randomized trial is warranted to determine its efficacy. 

## Conclusions

In a series of patients older than 70 years with LSS, significant improvements in pain were observed with minimally invasive decompressive surgery and a graded postoperative physiotherapy regimen focused on improving ambulation.
